# Hakim–Adams Syndrome: An Unusual Cause of Reversible Postoperative Coma

**DOI:** 10.3389/fmed.2016.00059

**Published:** 2016-11-21

**Authors:** Mohamed Saleh, Marine Bouex

**Affiliations:** ^1^Ales General Hospital, Ales, France; ^2^Montpellier University Hospital, Montpellier, France

**Keywords:** normal pressure hydrocephalus, postoperative coma, Hakim–Adams syndrome, general anesthesia, septic shock

## Abstract

We report the case of a 72-year-old patient presenting in our ICU with persistent postoperative coma in a context of recent unexplored neurological dysfunction. Detailed medical history taking from the patient’s family revealed he recently suffered from gait instability, urinary incontinence, and slight cognitive impairment. These constituted the clinical triad of normal pressure hydrocephalus syndrome. The presence of normal cerebrospinal fluid (CSF) pressure and distinctive radiological findings confirmed the diagnosis of normal pressure hydrocephalus or Hakims–Adams syndrome. After CSF volume subtraction (soustraction), the patient recovered a normal level of consciousness and was successfully weaned from mechanical ventilation. Normal pressure hydrocephalus should be included in the differential diagnosis of delayed postoperative arousal, especially in the elderly.

Normal pressure hydrocephalus or Hakims–Adams syndrome was first described in 1965, and the Hakim’s triad of gait instability, urinary incontinence, and dementia is mandatory for its correct diagnosis (1).

We present a 72-years-old male patient admitted into our ICU after surgical treatment of intestinal obstruction. Surgical procedure consisted of resection of a small intestinal necrotic volvulus.

The patient had no past medical history except for acute appendicitis several years ago and a recent history of unexplained gait instability and urinary incontinence.

He had no history of medication intake.

Surgery was uneventful and lasted for nearly 2 h. During the perioperative period, the patient presented with septic shock related to small intestine necrosis.

In addition to surgical resection of the necrotic volvulus, medical treatment consisted of short course of antibiotic therapy by amoxicillin–clavulanic acid combination for 5 days and circulatory support by small doses (0.2 μg/kg/min) of norepinephrine for 2 days during the postoperative period.

On ICU admission, the patient was kept on mechanical ventilation and required sedation.

His arterial blood pressure was 125/70 mmHg, heart rate 100 beats/min, oxygen saturation 100% (PaO_2_/FIO_2_ ratio was 400), body temperature 36°C, and blood sugar level 1.5 g/l. The patient’s Glasgow coma scale (GCS) was 3 (E1V1M1) on admission.

Standard biochemical blood analysis showed normal renal and liver function tests as well as normal arterial blood gases values. Blood lactate level was normal.

Complete blood count showed mild hyperleukocytosis.

No microorganisms grew in blood or peritoneal fluid specimen cultures.

Because of the patient’s old age, we stopped administration of anesthetic drugs (propofol and fentanyl) on the next day after ICU admission to hasten weaning from mechanical ventilation.

Seven days later, the patient presented with persistent and unexplained altered level of consciousness hindering weaning from mechanical ventilation.

Neurological assessment showed the patient was still comatose, and his GCS was 7 (E1V1M5). There was no motor deficit. Naloxone test was also attempted to exclude residual fentanyl impregnation, and residual drug induced muscle paralysis was clinically excluded since the patient showed normally oriented four limbs movements in response to painful stimuli.

Electroencephalogram showed diffuse non-specific weak cerebral activity with no signs of seizures.

We completed laboratory investigations by monitoring blood ammonia, thyroid hormones, and morning cortisol levels. All of which were within normal limits.

Brain CT showed diffuse periventricular hypodensities first diagnosed as age-related leukoaraiosis with no obvious signs of recent ischemic or hemorrhagic cerebrovascular accident. Careful examination of brain CT showed lateral ventriculomegaly, which was disproportionate to the age-related cortical cerebral atrophy (Figure 1). In addition to disproportionate ventriculomegaly, brain MRI showed also periventricular hyperintensities on T2-weighted sequences suggesting transependymal cerebrospinal fluid (CSF) resorption (Figure 2). Diagnosis of normal pressure hydrocephalus was then suspected.

**Figure 1:**
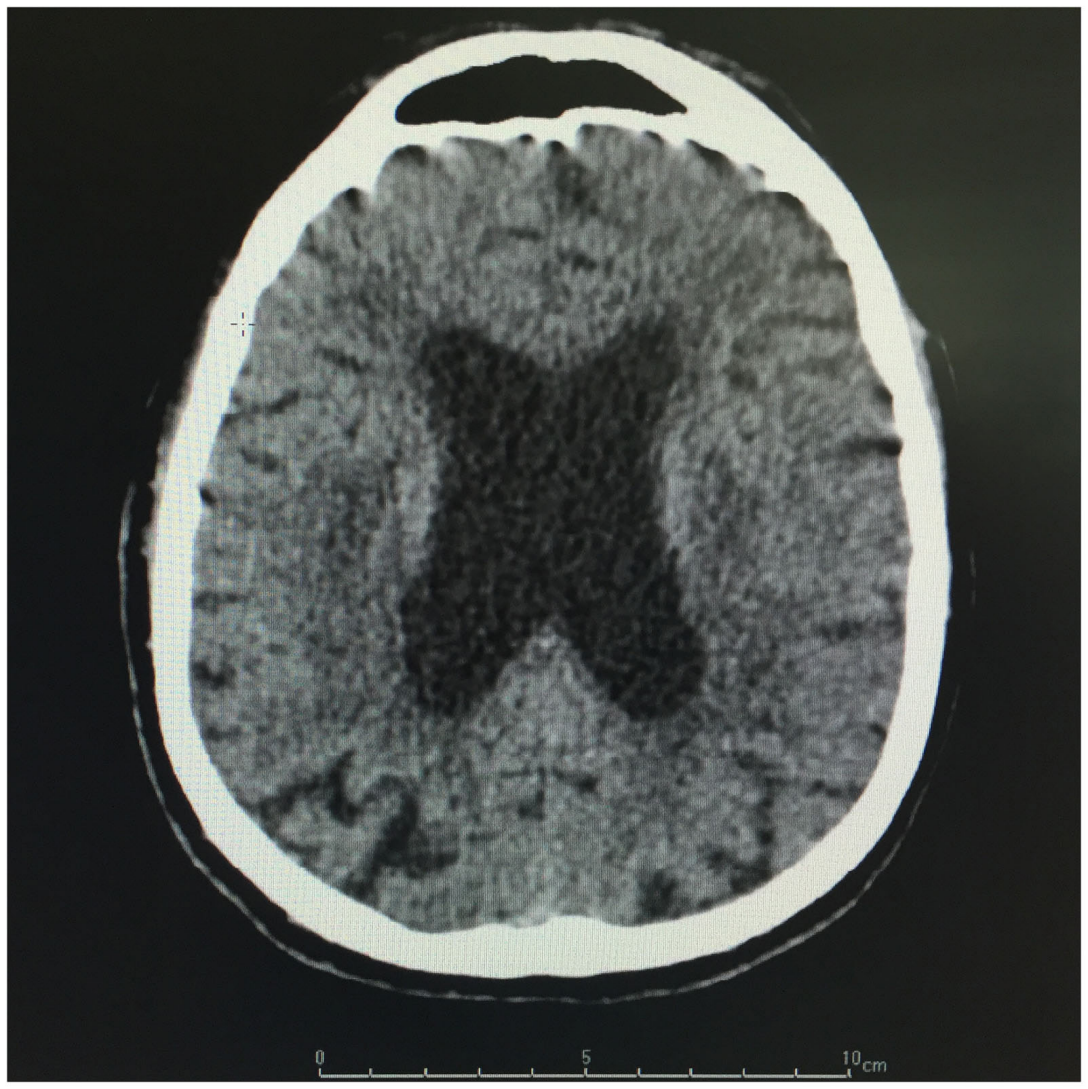
**Brain CT: Lateral ventriculomegaly and periventricular hypointensities**.

**Figure 2:**
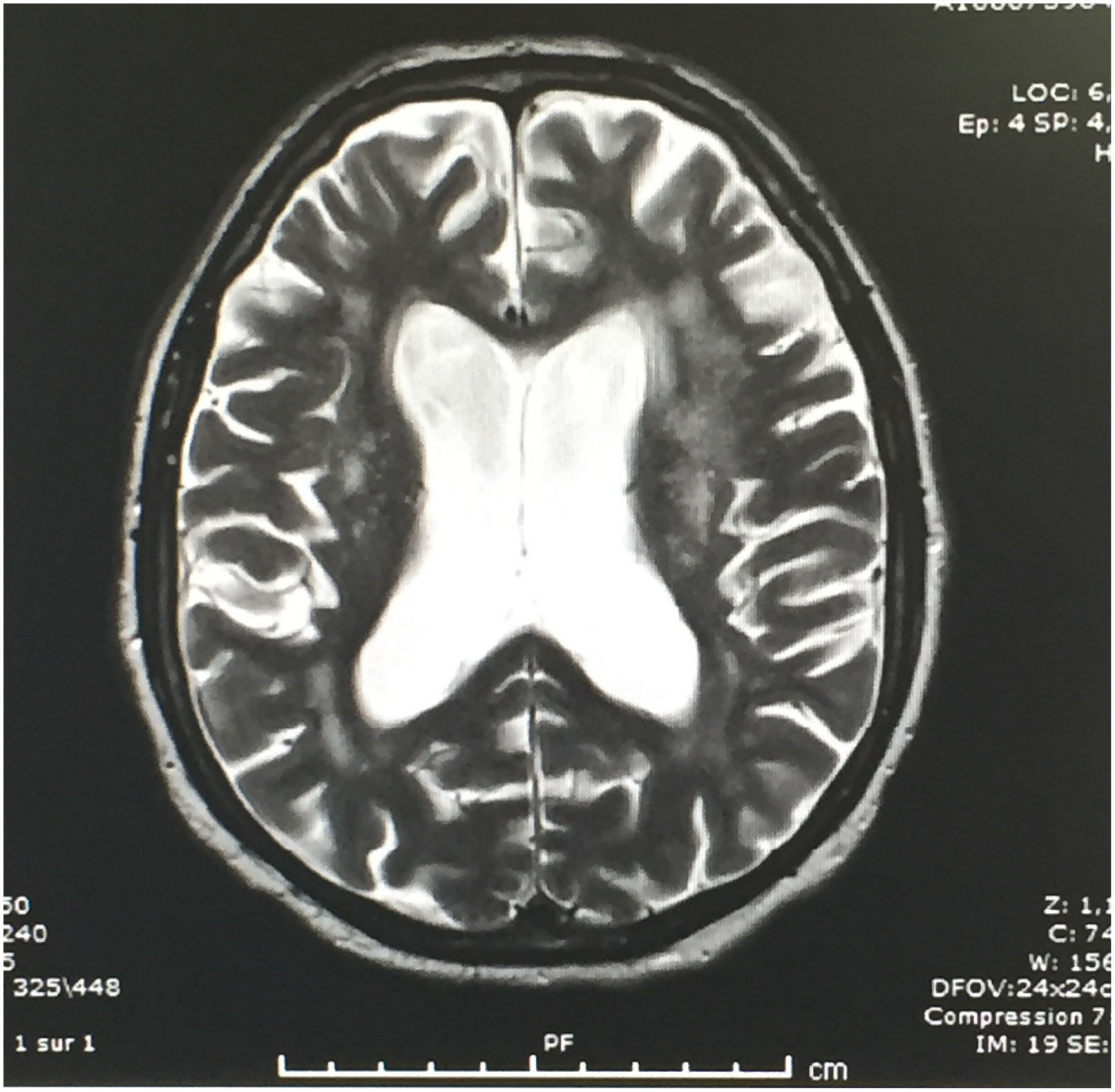
**T2-weighted brain MRI: Note periventricular hyperintensities in relation with CSF resorption**.

We performed a CSF tap-test and drained a CSF volume of 50 ml according to Wikkelsö (2). CSF opening pressure was 15 mmHg, and CSF analysis yielded normal cellular and biochemical results. CSF fluid bacterial culture remained negative as well as herpes simplex virus polymerase chain reaction test.

The patient regained full consciousness nearly 2 h after CSF drainage (GCS 15) and was successfully weaned from mechanical ventilation. By careful history taking, the patient’s family reported he suffered from gait instability, urinary incontinence, and memory loss many months before surgery. Clinical, radiological, and CSF diagnostic features of Hakim–Adams syndrome were thus fulfilled (2).

Because of lack of past medical history of neurological disease or brain trauma, the patient’s condition was diagnosed as idiopathic normal pressure hydrocephalus (iNPH), and he was then admitted in neurology department for shunt surgery.

Before attempting shunt surgery, some differential diagnosis of unexplained postoperative coma/drowsiness had to be excluded, these are listed as follows:

– Shock induced ischemic or post anoxic encephalopathy was the main differential diagnosis. Neither the clinical severity of the shock nor the lactate level provided strong evidence for post anoxic encephalopathy. Moreover, absence of signs of ischemic brain injury on brain imaging and rapid reversibility of the clinical picture argued against this hypothesis.
– Age-related neurodegenerative disorders (e.g., Alzheimer or Binswanger disease) were the most challenging differential diagnosis. Detailed history taking, brain imaging, and rapid clinical improvement after CSF volume subtraction (soustraction) argued against these diagnoses.
– Drugs/metabolic disorders-induced coma or subclinical seizures were the next differential diagnosis in this setting. The abovementioned clinical tests and investigations excluded these etiologies.
– Finally, the occurrence of primary central nervous system disease – notably herpes simplex virus encephalitis – has been recently reported in the postoperative period of some major surgical procedures (3). This diagnosis was excluded by CSF fluid specimen analysis.

## Discussion

Our findings suggest iNPH might be a cause of reversible coma especially in the ICU setting. This observation was previously supported by Tsai and coworkers who reported a case of reversible postoperative drowsiness and altered consciousness in a 71-year-old male patient suffering from undiagnosed iNPH and successfully treated by shunt surgery (4). In their observation, the authors incriminated anesthetic drugs as a trigger of long-lasting postoperative drowsiness and altered consciousness in this setting.

On the other hand, recent data suggested that the occurrence of low cerebral blood flow during iNPH clinical course might be responsible for the observed neurological dysfunction (5).

Strong correlation has also been found between clinical improvement and postoperative cerebral perfusion changes after shunt surgery (6), which remains a milestone in iNPH treatment.

According to these data, one might suppose anesthetic drugs and/or circulatory failure might worsen iNPH clinical features – leading sometimes to coma in the critical care setting.

In our case, both circulatory failure and anesthetic drugs administration could have played a role in worsening the clinical presentation of the disease.

It is also noteworthy to emphasize the role of brain MRI in this setting where transependymal CSF resorption might be mistaken for age-related white matter changes or leukoaraiosis on brain CT imaging. MRI T2-weighed sequences are of great help for correct diagnosis.

In selected cases, shunt surgery might be a good treatment option for this condition, but neurological dysfunction reversibility and the probability of long-term shunt responsiveness should be assessed by CSF tap-test before attempting such procedure (2).

Finally, one should also keep in mind that some clinical features and radiological findings of iNPH, like cognitive impairment and ventriculomegaly, might be shared by other diseases like Alzheimer disease and subcortical dementia (7). CSF drainage or shunt surgery in these cases would be useless and sometimes harmful. Consequently, all procedures aiming to reduce CSF volume should be preceded by careful medical history taking and brain imaging notably MRI T2-weighed sequences.

Although alteration in neurological and neuropsychological capacities – including level of vigilance – in patients suffering from iNPH has been well documented (8), the real impact of the disease on arousal after general anesthesia or in the critical care setting is unknown. Lack of data concerning this issue might be due to both old age and poor medical condition of the population of patients suffering from the disease. In fact, in these cases many confounding factors especially neurodegenerative disorders might be incriminated as causes of delayed or altered arousal.

Case series or reports are useful tools to better describe iNPH in the critical care setting and to increase ICU physicians’ awareness of this reversible and – probably underestimated – cause of altered consciousness in the ICU setting.

## Author Contributions

MS was the referent doctor of the patient and the author of the manuscript. MB was the junior doctor in charge and made review research.

## Conflict of Interest Statement

The authors declare that the research was conducted in the absence of any commercial or financial relationships that could be construed as a potential conflict of interest. The reviewer JR-F and handling Editor declared their shared affiliation, and the handling Editor states that the process nevertheless met the standards of a fair and objective review.
